# Citrus Essential Oil Nanoemulsions Mitigate Cardiac, Hepatic, and Pancreatic Injury in High‐Fat Obese Rats via Adiponectin/SIRT1/Nrf2 Signaling Pathways

**DOI:** 10.1155/jnme/3343221

**Published:** 2026-05-30

**Authors:** Amira Mira, Yhiya Amen, Abdelaziz M. Hussein, Abdullah Haikal, Mahmoud Elshal, Dalia Shaheen, Elsayed A. Eid, Ahmed Magdy Aly, Mennatullah A. M. Hussein, Abdelrahman H. K. Moustafa, Amgad E. Salem, Amal F. Soliman

**Affiliations:** ^1^ Department of Pharmacognosy & Pharmaceutical Chemistry, College of Dentistry & Pharmacy, Buraydah Colleges, Buraydah, 51418, Saudi Arabia; ^2^ Department of Pharmacognosy, Faculty of Pharmacy, Mansoura University, Mansoura, 35516, Egypt, mans.edu.eg; ^3^ Department of Medical Physiology, Mansoura Faculty of Medicine, Mansoura University, Mansoura, 35516, Egypt, mans.edu.eg; ^4^ Department of Medical Physiology, Faculty of Medicine, Delta University for Science and Technology, Gamasa, 11152, Egypt, deltauniv.edu.eg; ^5^ Department of Pharmacology and Toxicology, Faculty of Pharmacy, Mansoura University, Mansoura, 35516, Egypt, mans.edu.eg; ^6^ AlGhad College for Applied Medical Sciences, Najran, 66243, Saudi Arabia; ^7^ Department of Medical Biochemistry, Mansoura Faculty of Medicine, Mansoura University, Mansoura, 35516, Egypt, mans.edu.eg; ^8^ Department of Internal Medicine and Endocrinology, Faculty of Medicine, Delta University for Science and Technology, Gamasa, 11152, Egypt, deltauniv.edu.eg; ^9^ Faculty of Medicine, Mansoura University, Mansoura, 35516, Egypt, mans.edu.eg; ^10^ Department of Pharmaceutics, Faculty of Pharmacy, Mansoura University, Mansoura, 35516, Egypt, mans.edu.eg; ^11^ Department of Pharmacognosy, Faculty of Pharmacy, Mansoura National University, Gamasa, 7731168, Egypt

**Keywords:** adiponectin/SIRT1/Nrf2, *C. aurantiifolia*, *C. japonica*, *C. limon*, nanoemulsions, obesity

## Abstract

**Background:**

*Citrus* oils (COs) are consumed as herbal tea in traditional medicine to ameliorate the obesity‐induced consequences. *Citrus limon* oil (CLO), *C. aurantiifolia* oil (CAO), and *C. japonica* oil (CJO) are widely dispersed for their medicinal properties. However, it is yet unknown how these oils work to reduce obesity. This work investigates the potential beneficial effects of the nanoemulsions (NEs) of *Citrus* essential oils (EOs) on hepatic steatosis and cardiac and pancreatic injury in high‐fat diet (HFD)–induced obese rats and the mechanisms behind these effects.

**Methods:**

EOs were formulated as NEs using the ultrasonic emulsification method. The three NEs (150 mg/kg) were orally and separately administered along with the standard metformin (500 mg/kg) for 30 days after prior induction of obesity by HFD for a period of 12 weeks.

**Results:**

Treatments with NEs improved HFD‐induced obesity with different potentials. The NE of CJO represented more cytoprotective and antisteatotic activities. It resulted in a preferential reduction of body weight and serum levels of glucose and lipids, as well as tissue injury and oxidative stress biomarkers that were accompanied by improved tissue architectures. At the molecular level, it upregulated the antioxidants Nrf2, HO‐1, and SIRT1. It also increased the levels of adiponectin while decreasing visfatin. Furthermore, it downregulated the apoptotic executor, active caspase‐3.

**Conclusions:**

The CJO NEs have shown strong promise in lowering obesity‐induced hepatic steatosis and mitigating cardiac and pancreatic injury via hypoglycemic, antidyslipidemic, cytoprotective, antioxidant, and antiapoptotic activities. These beneficial effects are collectively associated with modulating the adiponectin/SIRT1/Nrf2 signaling cascade.

## 1. Introduction

Obesity, which is defined as a body mass index (BMI) exceeding 30 kg/m^2^, leads to an excessive ectopic fat deposition (steatosis) of different organs with subsequent severe metabolic and clinical implications, including metabolic syndrome, Type 2 diabetes, and cardiovascular diseases [1, 2]. Obesity is associated with chronic low‐grade inflammation, known as metaflammation, which is one of the main underlying pathophysiological features of metabolic disorders, often triggered by the associated excessive fat accumulation [3]. Besides inflammation, redox imbalance, which is due to elevated oxidative stress and a repressed antioxidant defense system, is common in obesity. Although obesity‐associated complications might vary from one to another, the consequences are similar for all due to the presence of oxidative stress and inflammation in all obese patients [4]. Oxidative stress in accumulated fat was reported to mediate the obesity‐associated development of metabolic syndrome, which is correlated with the dysregulated production of tissue‐derived regulatory molecules called adipocytokines, mainly adiponectin and visfatin [4].

Essential oils (EOs) are composed of natural combinations of terpenes, particularly monoterpenes and sesquiterpenes. In the field of pharmaceutics, they are primarily used for aromatherapy and enhancing the sensory attributes of pharmacological treatments. Various traditional systems worldwide utilize EOs to remedy a variety of health issues [5–9]. Numerous in vivo and in vitro investigations have demonstrated the antiobesity properties of EOs through the regulation of lipolysis and lipogenesis, the induction of browning, and the modulation of certain adipocytokines [10].

*Citrus* fruits are considered as a highly antioxidant and nutritious source due to their contents of flavonoids, vitamin C, fibers, and pectins. Traditionally, they are used daily by patients who are following strict diets to control obesity [11]. EOs of the Rutaceae family, especially some of the *Citrus* species, were previously and separately investigated for in vitro and in vivo antiobesity effects [12–16]. However, the antisteatotic effects of the EOs of the *Citrus* species on the dietary obesity‐induced steatosis in different organs, including the liver, heart, and pancreas, have not yet been studied. The formulation of EOs in nanoemulsions (NEs) is one of the recent, advanced strategies to overcome the major drawbacks of EOs, such as hydrophobicity, high first‐pass metabolism, which limits bioavailability, and disagreeable taste of some EOs [17]. Recently, it has been reported that NEs enhanced the antiobesity potential of the original EOs [18]. Accordingly, this study aimed to explore and compare the antisteatotic effects of the NEs of three *Citrus* EOs: *Citrus limon* oil (CLO)*, C. aurantiifolia* oil (CAO), and *C. japonica* oil (CJO) on different organs in high‐fat diet (HFD)‐induced obesity in rats, and elucidate the underlying molecular mechanisms.

## 2. Methods

### 2.1. Plant Materials

The aerial parts of *C. limon*, *C. aurantiifolia*, and *C. japonica* were gathered at the fruiting stage in November 2022, from the gardens of Mansoura University in Egypt. Dr. Ibrahim Mashaly, a Professor of Plant Ecology at the Faculty of Science, Mansoura University, confirmed the identity of the plants. The Department of Pharmacognosy at Mansoura University housed voucher specimens with the codes CL‐11‐2022, CA‐11‐2022, and CJ‐11‐2022. The plants were collected according to IUCN Policy Statement on Research Involving Species at Risk of Extinction and the Convention on International Trade in Endangered Species of Wild Fauna and Flora.

### 2.2. Hydrodistillation of the EOs

Fresh aerial parts of *C. limon*, *C. aurantiifolia*, and *C. japonica* (each weighing 250 g) were ground into small fragments and hydrodistilled for 3 h with Clevenger‐type apparatus to obtain clear yellow oils. These oils were collected, dehydrated using anhydrous sodium sulfate, and stored in sealed vials at low temperatures for GC/MS analysis and biological studies.

### 2.3. Gas Chromatography–Mass Spectrometry (GC/MS) Analysis

The GC/MS analysis was performed using a Thermo Scientific Trace GC‐ISQ mass spectrometer equipped with an A3000 autosampler and a TG‐5MS capillary column measuring 30 m in length, 0.25 mm in inner diameter, and 0.25 m in film thickness. Between 50°C and 280°C, the temperature was planned to be increased in the gradient mode (10°C/minute). Temperature of the source: 200°C; temperature at the interface: 220°C; temperature of the injector: 220°C. The mass spectrometer was set to the EI mode with an energy of 70 eV. In the splitless mode, 1 μL of the diluted sample was injected using a mass scan of 50–600 amu. Helium served as the carrier gas at a rate of 1 mL/min. To identify the oil components based on their retention indices, a mixture of a homologous series of *n*‐alkanes (C8–C28) was promptly introduced into the GC injector according to the previously mentioned temperature program, allowing for the calculation of each molecule’s retention index. All component retention indices were determined using the method developed by Van Den Dool [19]. Further confirmation of the identity of the components was provided by comparing the mass spectral fragmentation patterns and the base peak of the component with those documented in the literature [20] or in the mass spectral databases NIST and the ChemStation data system.

### 2.4. Preparation of NEs

NEs of the three oils, CLO, CAO, and CJO, were prepared by the ultrasonic emulsification method. The ultrasonic emulsification method has advantages over other preparation methods as it results in economical, efficient production of NEs of high stability and does not require a high concentration of surfactant so it produces smaller particle‐sized NEs with no toxicity of surfactant [21]. NEs were prepared using 10% (w/w) oil, 20% (w/w) tween 20 (T20, Sigma‐Aldrich, St. Louis, MO, USA) as surfactant, and 70% (w/w) deionized water as aqueous phase. First, T20 was dissolved in phosphate‐buffered saline (PBS, pH 7.4), and then, it was slowly added to the calculated amount of each oil under magnetic stirring at 400 rpm (Jenway 1100, Hotplate & Stirrer, Jenway) for 10 min at ambient temperature (25°C) to obtain the crude emulsion (CE). Second, the CE was probe‐sonicated (Sonics Vibra‐cell, Sonic & Materials, USA) with 20 kHz frequency and 750 W power using the pulsed mode (1 s on, 1 s off) at 60% amplitude for 8 min. Finally, the samples were placed in an ice water bath during the sonication process, and then, the samples were stored protected from light at 25°C.

### 2.5. Particle Size, Polydispersity Index, Zeta Potential, and Micromorphology

To avoid multiple scattering effects, samples were diluted with deionized water (1:2, v/v) before using the Malvern dynamic light scattering instrument Zetasizer (Nano ZS90, Malvern Instruments Ltd., UK) to measure the particle size, polydispersity index, and zeta potential of NEs. After that, NEs were mounted on a carbon‐coated grid and air‐dried, and the samples were observed and photographed with a transmission electron microscope (TEM, JEM‐2100F electron microscope, JEOL Ltd., Tokyo, Japan).

### 2.6. Experimental Animals

Thirty‐five male Sprague–Dawley rats (180 ± 20 g) were utilized. Rats were placed in plastic cages at a temperature of 25°C, allowed free food and water, and sustained on a 12‐h light/dark cycle. The experimental procedures were carried out at the animal house, Faculty of Pharmacy, Mansoura University, after the approval of the Animal Care and Use Committee MU‐ACUC (Code: PHARM.R23.06.23).

### 2.7. Experimental Design

After acclimatization, rats were randomly divided into two groups based on assigned diets: standard rat chow (normal group, *n* = 5, Group 1) and a HFD (MP Diets, USA) for 12 weeks (HFD group, *n* = 30, Group 2). The body weight of each rat in both groups was recorded twice weekly to ensure the development of obesity in the HFD group. After 12 weeks of the assigned diet, rats of Group 2 were subdivided into six groups (*n* = 5) as follows: HFD group: rats were continually fed on HFD for other 30 days; vehicle group: rats were continually fed on HFD plus daily oral administration (600 μL/day) with the NE vehicle (Tween 20+ DW; 20: 80 v/v %) without drugs for 30 days; metformin group: rats fed on HFD plus daily oral dose of metformin (500 mg/kg) as a standard drug for 30 days [22, 23]; CLO group: rats were continually fed on HFD plus daily oral administration (600 μL/day) with NE of CLO (150 mg/kg) for 30 days; CAO group: rats were continually fed on HFD plus daily oral administration (600 μL/day) with NE of CAO (150 mg/kg) for 30 days; and CJO group: rats were continually fed on HFD plus daily oral administration (600 μL/day) with NE of CJO (150 mg/kg) for 30 days. The selected doses of the NE treatments were determined based on our preliminary previous studies by Ragab et al. [24] and Sabry et al. [25].

### 2.8. Preparation of Samples

By the end of the experiment, the rats were fasted overnight and anaesthetized by intraperitoneal injection of thiopental (50 mg/kg) [26], and blood samples were collected via cardiac puncture. Then, the rat’s abdomen and thorax were rapidly opened, and the heart, liver, and pancreas were isolated. Then, they were cut into parts, and the first part of each was placed in formaldehyde solution (10% v/v) for preparation of paraffin blocks for histopathological and immunostaining examination, and the second part was stored at liquid nitrogen for polymerase chain reaction (PCR) study, whereas the third part was used for tissue homogenate preparation in PBS, pH 7.4. The homogenate was centrifuged at 4000 rpm for 15 min at 4°C, and then, the supernatant was stored at −20°C for subsequent analysis.

### 2.9. Determination of Body Weight Change and Relative Organs’ Weights

Rats of all groups were weighed at the start and at the end of the experiment, and the initial and final body weights were recorded. The percentage change in body weight was calculated according to the following equation: [(final body weight − initial body weight)/final body weight] × 100 [27].

### 2.10. Determination of Blood Glucose, Lipid Profile, and Serum Enzyme Biomarkers

Blood samples were analyzed for fasting blood glucose levels using an Accu‐Chek Active glucometer and test strips supplied. The lipid profile was addressed by measuring triglycerides (TGs) and total cholesterol concentrations by the colorimetric method using commercially available kits (SPIN REACT, Spain) according to the manufacturer’s instructions. In addition, serum levels of enzymes were determined as injury biomarkers, including cardiac enzyme [creatine kinase‐myocardial band (CK‐MB)], liver enzyme [alanine aminotransferase (ALT)], and pancreatic enzyme (serum amylase), using commercially available kits according to the manufacturer’s instructions.

### 2.11. Determination of Serum Visfatin and Adiponectin

The quantitative determination of serum adiponectin and visfatin was performed using specific ELISA kits (Abcam, MA, USA, Cat# ab239421 and ab267799, respectively) according to the manufacturer’s instructions.

### 2.12. Determination of Redox State in Heart, Liver, and Pancreas Tissues

The lipid peroxidation biomarker malondialdehyde (MDA) content as well as the antioxidant enzymes reduced glutathione (GSH) and catalase (CAT) activities was determined in tissue homogenates to evaluate the redox state, through colorimetric methods using the corresponding kits (Bio‐Diagnostics, Giza, Egypt) and following the manufacturer’s instructions.

### 2.13. Histopathological Examination

Tissue sections (4 μm thickness) were prepared from paraffin blocks and then stained using hematoxylin and eosin (H&E). Slides were examined for signs of damage: deranged cells, uneven cytoplasm distribution, edema, inflammatory cell infiltrate, cell rupture, irregular nuclei, and interstitial hemorrhage using light microscope (Leica DM500 with camera Leica ICC50HD) with software Camera software LEICA Application Suite (LAS) EZ, Version 3.1.1 (Physiology and Biotechnology Laboratory, Department of Animal Production, Faculty of Agriculture, Mansoura University). For each rat, three nonoverlapping tissue sections were assessed, and within each section, five random fields were examined. Quantitative measurements from all analyzed fields were averaged to obtain a single representative value per animal, and these animal‐level means were used for statistical analysis. Scoring of heart tissue lesions was performed according to Ref. [28] by summation of myonecrotic, inflammatory, and hemorrhagic changes as follows: myonecrosis (0, none; 1, rare to minimal; 2, mild to moderate and focal to multifocal; and 3, severe and diffuse), inflammation (0, none; 1, few; 2, focal to multifocal; and 3, coalescing and diffuse), and hemorrhage (0, none; 1, rare; 2, minimal to mild; and 3, severe). Regarding liver tissues, a scoring system that considers the summation of steatosis, necrosis, and inflammation was adopted [29], as follows: steatosis (0, none; 1, few intracytoplasmic vacuoles; 2, mild to moderate intracytoplasmic vacuoles; and 3, many intracytoplasmic vacuoles), necrosis (0, none; 1, rare necrotic cells; 2, few minimal to mild necrotic cells; and 3, many moderate to severe multifocal to coalescing necrotic cells), and inflammation (0, none; 1, focal inflammatory aggregates; 2, multifocal inflammatory foci; and 3, many coalescing inflammatory foci). Otherwise, the used scoring system for pancreatic tissue lesions was based on Ref. [30], as follows: necrotic changes (0, none; 1, rare necrotic cells; 2, minimal to mild acinar necrosis; and 3, moderate to severe acinar necrosis), inflammatory changes (0, none; 1, few; 2, moderate; and 3, severe), and vascular changes (0, none; 1, few rare interlobular edema; 2, mild to moderate interlobular edema; 3, moderate to severe interlobular edema).

### 2.14. Real‐Time PCR

In this experiment, about 50–100 mg of each tissue was homogenized in 1 mL of TRIzol to obtain total RNA according to the manufacturer’s instructions (Invitrogen Corporation, NY, USA). Reverse transcription was performed using 1 μg total RNA and a cDNA kit (high‐capacity cDNA archive kit). The sequence of the primers of the tested genes was as follow: nuclear factor‐erythroid 2‐related factor 2 *(Nrf2)*, forward: 5′‐GCTATTTTCCATTCCCGAGTTAC‐3′, reverse: 5′ATTGCTGTCCATCTCTGTCAG‐3′; heme oxygenase‐1 (HO‐1), forward: 5′‐CTTTCAGAAGGGTCAGGTGTC‐3′, reverse: 5′‐TGCTTGTTTCGCTCTATCTCC‐3′; *GAPDH* forward: 5′‐TATCGGACGCCTGGTTAC‐3′, reverse: 5′‐CTGTGCCGTTGAACTTGC‐3′.

#### 2.14.1. Immunohistochemical Examination

Heart, liver, and pancreas sections were deparaffinized, rehydrated, washed, immersed in 3% hydrogen peroxide, and then digested with pepsin for antigen retrieval. The primary rabbit polyclonal anticaspase‐3 (Cell Signaling Technology Cat. no. 9664) was diluted at 1:50, while the primary goat polyclonal anti‐Sirt‐1 (Santa Cruz sc‐19857, Santa Cruz, CA, USA) was diluted at 1:200. After the blocking of unspecific binding by serum, the sections were incubated with primary antibodies against Sirt‐1 and caspase‐3 at 4°C overnight. The diaminobenzidine/peroxidase substrate was used to produce a brown‐colored signal. The sections were counterstained, dehydrated, cleared, and cover‐slipped. PBS was used to replace the primary antibody, and the adjacent sections were used as a negative control. The density of immunostaining was quantified as the percentage of tissue area occupied by positive staining using ImageJ software.

### 2.15. Statistical Analysis

Graphs were developed using GraphPad Prism 8. Parametric data (mean ± SE) were statistically analyzed by one‐way ANOVA, ensued by the Tukey–Kramer multiple comparison test. The histopathological scores were analyzed nonparametrically by the Kruskal–Wallis test. Statistical significance was considered at *p* < 0.05.

## 3. Results

### 3.1. Analysis of EOs

The three EOs were obtained as clear yellow oils lighter than water. D‐limonene is the major constituent of CLO and CAO, while *β*‐eudesmol is the major constituent of CJO (Figure 1, Table 1, and Supporting Tables S1–S3).

FIGURE 1Gas chromatograms of essential oils of (a) CLO (*Citrus limon*), (b) CAO (*Citrus aurantiifolia*), and (c) CJO (*Citrus japonica*).

**Figure:**
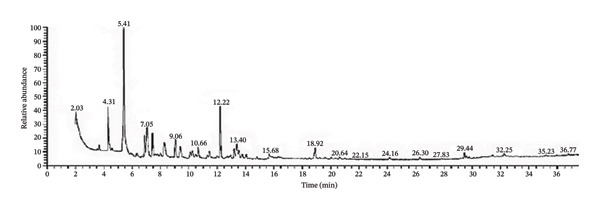
(a)

**Figure:**
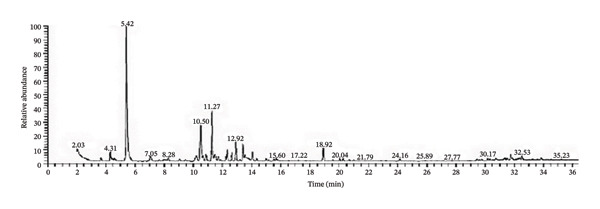
(b)

**Figure:**
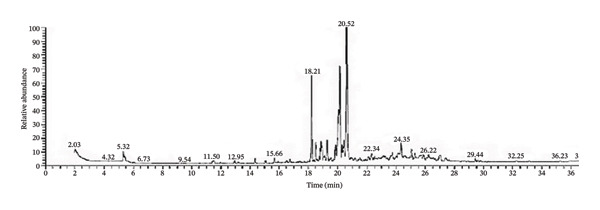
(c)

**TABLE 1:** Yield and comparative GC/MS analysis of the CLO, CAO, and CJO composition.

Parameter	CLO	CAO	CJO
Yield % (v/w)	0.7	0.8	0.3
*Chemical composition*			
‐ Number of identified compounds	20 (98.99%).	28 (99.9%).	35 (99.69%).
‐ Monoterpenes	Major (91.1%). ‐ Oxygenated monoterpenes present 48.22%. ‐ D‐limonene is major with 25.48%, 5‐caranol (10.85%), sabinene (8.43%) and linalool (7.60%).	Major (89.5%). ‐ Oxygenated monoterpenes present 52.16%. ‐ D‐limonene is major with 32.38%, neral (9.52%) and geraniol (12.92%).	Minor (4.4%). ‐ Oxygenated monoterpenes are absent. ‐ O‐cymene is a main compound (1.99%).
‐ Sesquiterpenes	Minor (3.32%). ‐ Oxygenated sesquiterpenes (3.32%). ‐ Caryophyllene oxide is a major (2.37%).	Minor (7.56%). ‐ Oxygenated sesquiterpenes represent 6.51% of the sesquiterpenes. ‐ Caryophyllene oxide is a major (3.36%).	Major (89.78%). ‐ Oxygenated sesquiterpenes represent 67.6% of the sesquiterpenes. ‐ β‐eudesmol (20.25%), β‐selinene (12.79%) and hinesol (12.62%).
‐ Diterpenes	—	—	Minor (3.18%). ‐ Oxygenated phytol (1.72%) and retinal (1.46%).

### 3.2. Characterization of NEs

NEs of CLO, CAO, and CJO were transparent liquids with an average droplet size of 142.47 ± 0.39 nm, 175.37 ± 4.61 nm, and 341.57 ± 10.35 nm, PDIs of 0.55 ± 0.004, 0.41 ± 0.01, and 0.37 ± 0.08, and zeta potentials of −0.63 ± 0.02, −2.52 ± 0.52, and −1.28 ± 0.14 mV, respectively. The nonionic nature of the surfactant (Tween 20), which makes up a significant portion of the NE, may be the cause of the NE’s tiny or almost neutral charge [31]. The droplets were nonaggregated, uniformly distributed, and had a spherical shape under the TEM (Figure 2). The average particle size of the samples indicated that the selected oils were in the nanosize range with a high surface area. The zeta potential measurement demonstrated that the CAO has a more negative charge than CLO and CJO. This could be due to the high content of aldehyde groups in the oil component, including citronellal, neral, and geranial, while the negative charge of CJO may be caused by the high content of alcoholic groups as it contains 14 alcoholic compounds; out of 35 components of the oil, the majority of them are spathulenol, β‐oplopanone, hinesol, and β‐eudesmol [32, 33].

FIGURE 2Transmission electron microscope photomicrographs of the nanoemulsions of (a) CLO (*Citrus limon*), (b) CAO (*Citrus aurantiifolia*), and (c) CJO (*Citrus japonica*).

**Figure:**
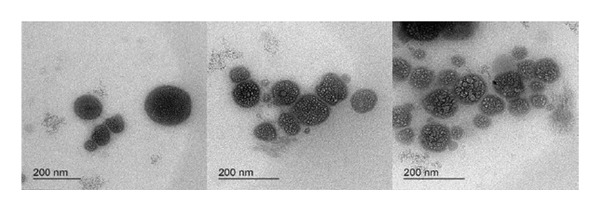
(a)

**Figure:**
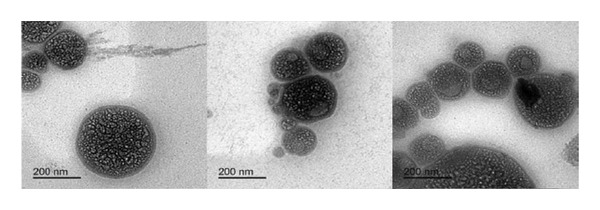
(b)

**Figure:**
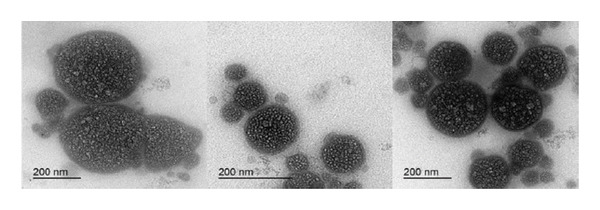
(c)

### 3.3. In Vivo Measurements

It was noticed for all the in vivo measurements that the vehicle (Tween 20+ DW; 20: 80 v/v %) used in the preparation of NEs of CLO, CAO, and CJO had no interfering effect on the activity of NEs.

#### 3.3.1. Changes in Body Weight and Levels of Blood Glucose, TGs, and Cholesterol in Obese Rats

As shown in Figure 3(a), the percentage change in body weight of rats significantly (*p* < 0.001) increased in the HFD and vehicle groups compared to the normal group, while treatment with either metformin or NEs of CLO, CAO, and CJO caused a significant (*p* < 0.001) reduction in the body weight change percentage compared to the HFD group. Notably, there was a significant difference in the body weight change between the CAO group and either the metformin (*p* < 0.001) or CJO (*p* < 0.05) groups. Otherwise, fasting blood glucose (Figure 3(b)), TGs (Figure 3(c)), and cholesterol (Figure 3(d)) levels were significantly (*p* < 0.001) increased in the HFD and vehicle groups compared to the normal group. Obviously, the blood glucose level was significantly decreased only in the metformin (*p* < 0.001) and CJO (*p* < 0.01) groups with no significant difference between them. Regarding TGs, their levels were significantly reduced in the metformin (*p* < 0.001) and the three EO groups (*p* < 0.01) compared to the HFD group. Meanwhile, the decrease in cholesterol levels in the treated groups was significant (*p* < 0.05) only in the metformin and CLO groups when compared to the HFD group.

FIGURE 3Effect of CLO (*Citrus limon*), CAO (*Citrus aurantiifolia*), and CJO (*Citrus japonica*) on (a) body weight, (b) fasting blood glucose, (c) serum triglyceride (TGs), and (d) serum total cholesterol in high‐fat diet (HFD)‐induced obesity in rats. Data are expressed as mean ± SEM. Significances are assigned as follows: ^∗^, compared to the normal control group; #, compared to the HFD group; $, compared to the vehicle group; @, compared to the metformin group; €, compared to the CLO group; ψ, the CAO group compared to the CJO group, using one‐way ANOVA followed by the Tukey–Kramer multiple comparison post hoc test.

**Figure:**
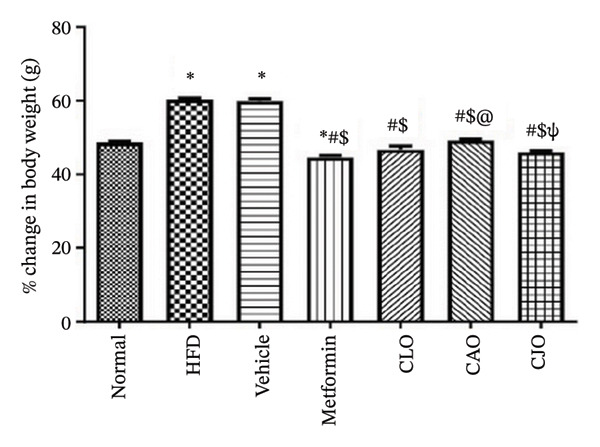
(a)

**Figure:**
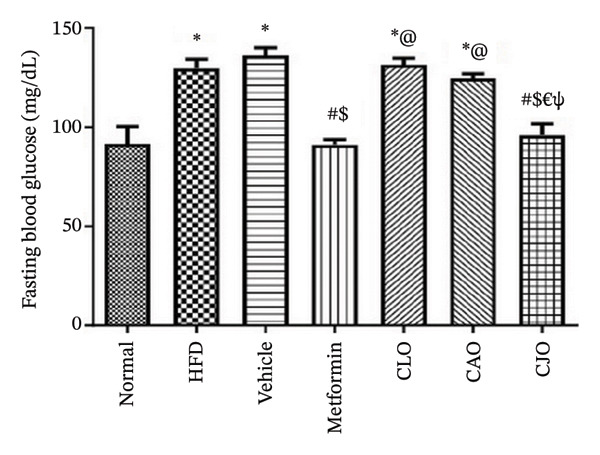
(b)

**Figure:**
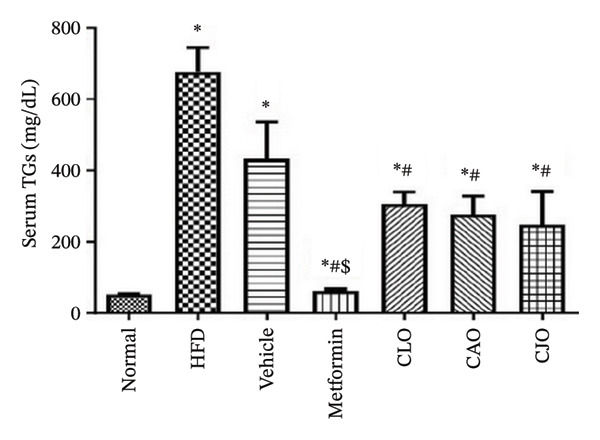
(c)

**Figure:**
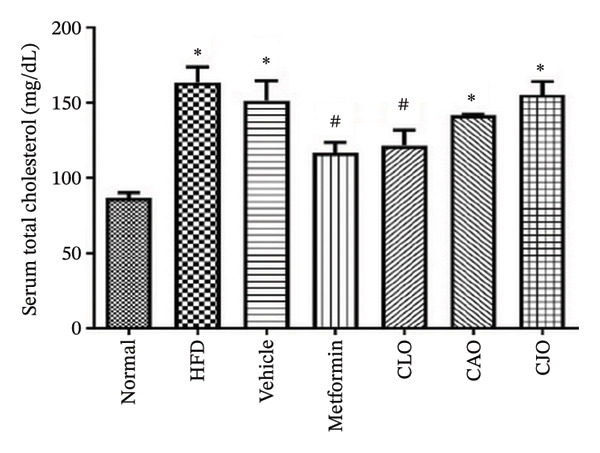
(d)

#### 3.3.2. Changes in Serum Biomarkers of Cardiac, Liver, and Pancreatic Functions in Obese Rats

The serum levels of the cardiac function marker CK‐MB [34], the liver function marker ALT [35], and the pancreatic function marker amylase enzyme [36] showed a significant rise in the HFD and vehicle groups compared to the normal group (Figures 4(a), 4(b), and 4(c), respectively). Meanwhile, the CK‐MB levels were significantly reduced in all the treatment groups compared to the HFD and vehicle groups with a significantly higher effect for CAO and CJO NEs than NE of CLO. Notably, the levels of CK‐MB were significantly (*p* < 0.05) lower in the CAO group than the metformin group. Meanwhile, only the CAO and CJO groups manifested a significant (*p* < 0.05 and 0.01, respectively) decline in ALT levels in comparison to the HFD and vehicle groups. Otherwise, a significant (*p* < 0.05) reduction in amylase enzyme levels occurred only in the metformin (*p* < 0.001) and CJO (*p* < 0.01) groups compared to the HFD group with no significant difference between their effects. Regarding CK levels, each of the metformin (*p* < 0.05), CAO (*p* < 0.01), and CJO (*p* < 0.001) groups displayed a significant decrease in its levels when compared with the HFD group.

FIGURE 4Effect of CLO (*Citrus limon*), CAO (*Citrus aurantiifolia*), and CJO (*Citrus japonica*) on serum (a) creatine kinase‐myocardial band (CK‐MB), (b) alanine aminotransferase, and (c) amylase in high‐fat diet (HFD)‐induced obesity in rats. Data are expressed as mean ± SEM. Significances are assigned as follows: ^∗^, compared to the normal control group; #, compared to the HFD group; $, compared to the vehicle group; @, compared to the metformin group; ^€^, compared to the CLO group, using one‐way ANOVA followed by the Tukey–Kramer multiple comparison post hoc test.

**Figure:**
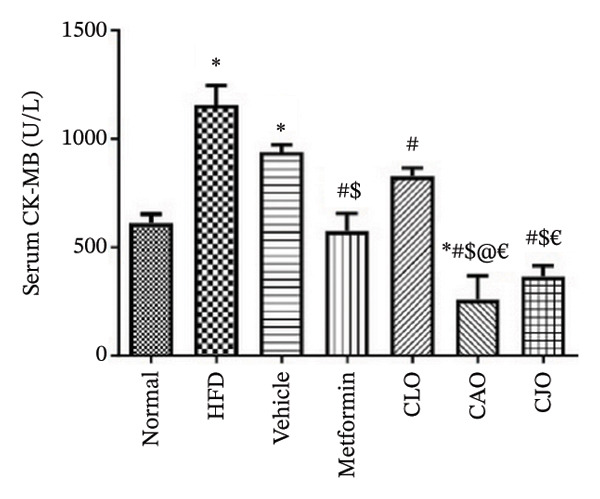
(a)

**Figure:**
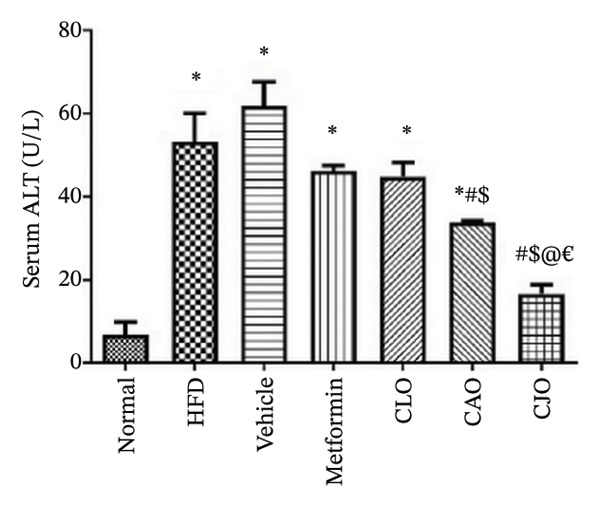
(b)

**Figure:**
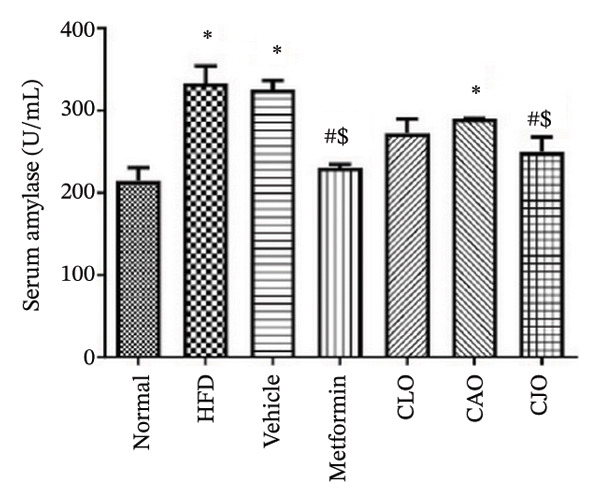
(c)

#### 3.3.3. Changes in Oxidative Stress Status in the Heart, Liver, and Pancreas in Obese Rats

The levels of the lipid peroxidation biomarker MDA in the heart, liver, and pancreas were significantly (*p* < 0.001) higher in the HFD and vehicle groups than in the normal control group (Figures 5(a), 5(b), and 5(c), respectively). Meanwhile, the oral consumption of the three NEs could efficiently and significantly (*p* < 0.001) reduce the MDA levels, with the NE of CJO being the most powerful one and showing no significant difference compared to metformin. On the other hand, the cardiac, hepatic, and pancreatic levels of antioxidants GSH (Figures 6(a), 6(b), and 6(c), respectively) and CAT (Figures 7(a), 7(b), and 7(c), respectively) were significantly (*p* < 0.001) reduced in the HFD and vehicle groups compared to the normal control group. Otherwise, CLO and CAO NE treatments were able to elevate cardiac GSH levels compared to the HFD and vehicle groups, whereas the NE of CJO treatment significantly elevated GSH levels in all examined tissues compared to the HFD and vehicle groups. Regarding the CAT enzyme, the three NE treatments significantly elevated its levels in all examined tissues compared to the HFD and vehicle groups, except for CAO NE in the liver. Notably, the CJO group recorded the highest levels of GSH and CAT compared to the CLO and CAO groups.

FIGURE 5Effect of CLO (*Citrus limon*), CAO (*Citrus aurantiifolia*), and CJO (*Citrus japonica*) on malondialdehyde (MDA) levels in (a) heart, (b) liver, and (c) pancreas in high‐fat diet (HFD)‐induced obesity in rats. Data are expressed as mean ± SEM. Significances are assigned as follows: ^∗^, compared to the normal control group; #, compared to the HFD group; $, compared to the vehicle group; @, compared to the metformin group; ψ, the CAO group compared to the CJO group, using one‐way ANOVA followed by the Tukey–Kramer multiple comparison post hoc test.

**Figure:**
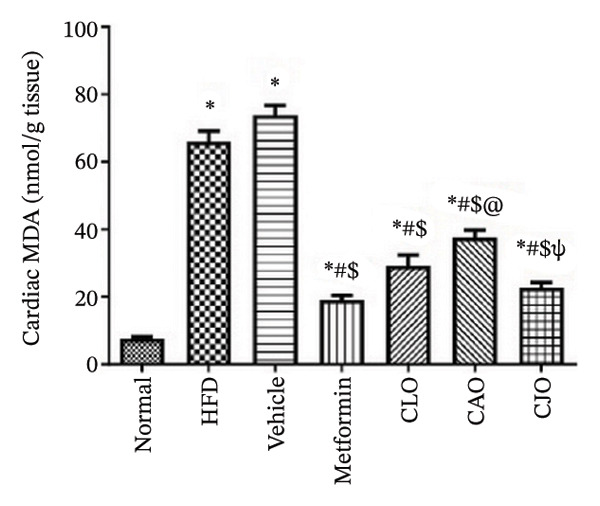
(a)

**Figure:**
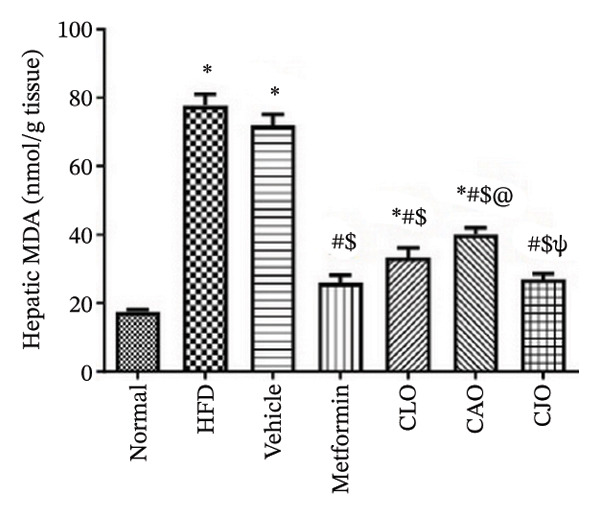
(b)

**Figure:**
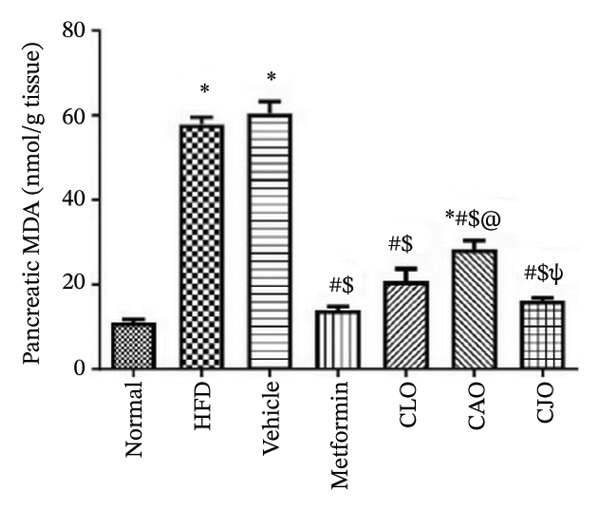
(c)

FIGURE 6Effect of CLO (*Citrus limon*), CAO (*Citrus aurantiifolia*), and CJO (*Citrus japonica*) on reduced glutathione (GSH) levels in (a) heart, (b) liver, and (c) pancreas in high‐fat diet (HFD)‐induced obesity in rats. Data are expressed as mean ± SEM. Significances are assigned as follows: ^∗^, compared to the normal control group; #, compared to the HFD group; $, compared to the vehicle group; @, compared to the metformin group; ψ, the CAO group compared to the CJO group, using one‐way ANOVA followed by the Tukey–Kramer multiple comparison post hoc test.

**Figure:**
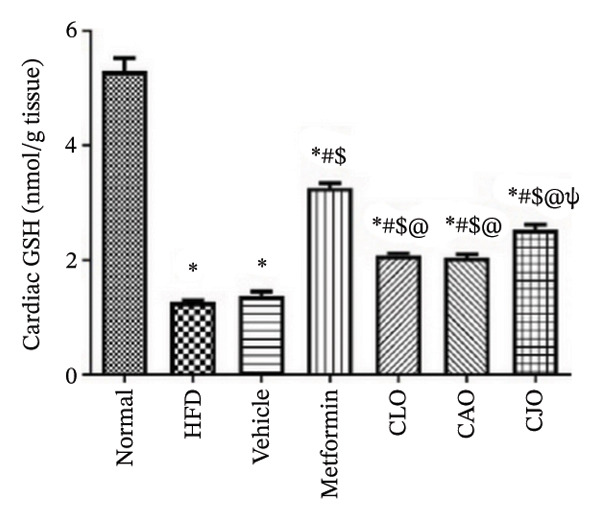
(a)

**Figure:**
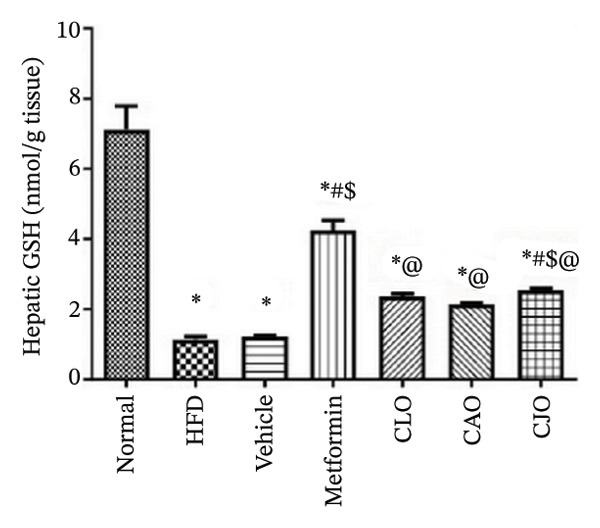
(b)

**Figure:**
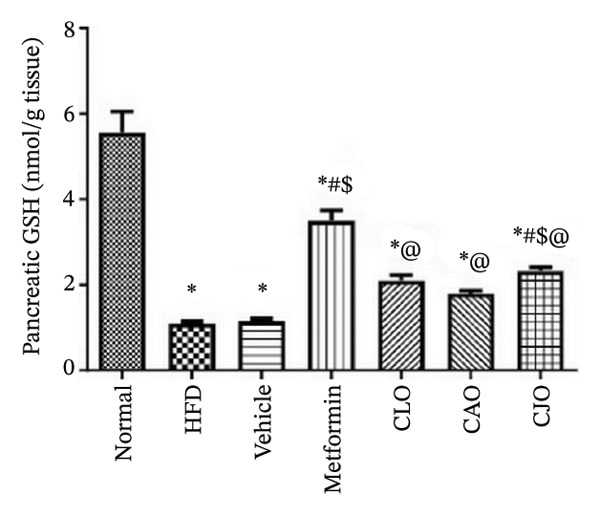
(c)

FIGURE 7Effect of CLO (*Citrus limon*), CAO (*Citrus aurantiifolia*), and CJO (*Citrus japonica*) on catalase activity in (a) heart, (b) liver, and (c) pancreas in high‐fat diet (HFD)‐induced obesity in rats. Data are expressed as mean ± SEM. Significances are assigned as follows: ^∗^, compared to the normal control group; #, compared to the HFD group; $, compared to the vehicle group; @, compared to the metformin group; ψ, the CAO group compared to the CJO group, using one‐way ANOVA followed by the Tukey–Kramer multiple comparison post hoc test.

**Figure:**
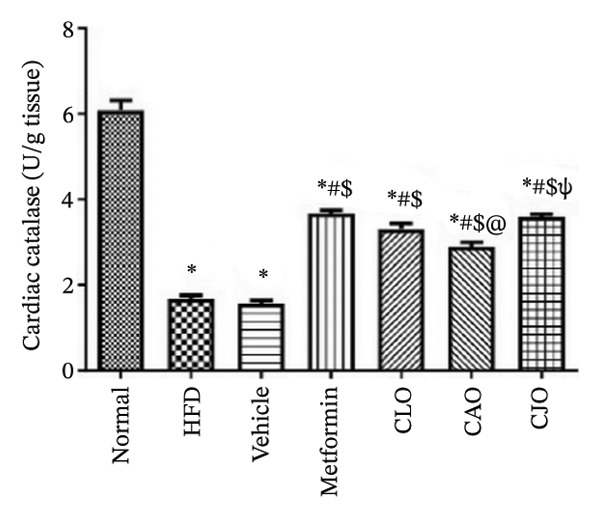
(a)

**Figure:**
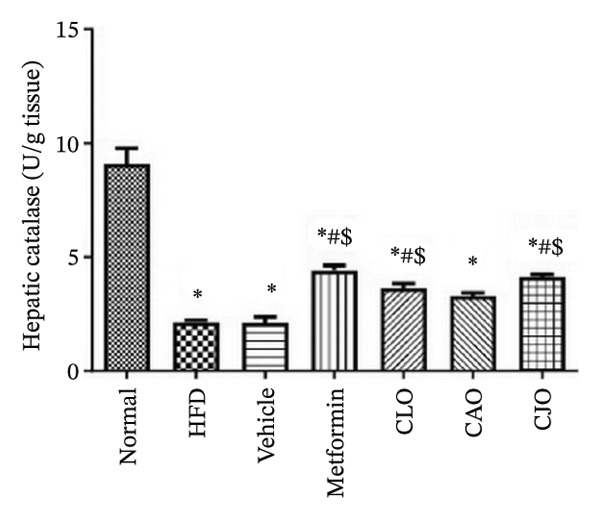
(b)

**Figure:**
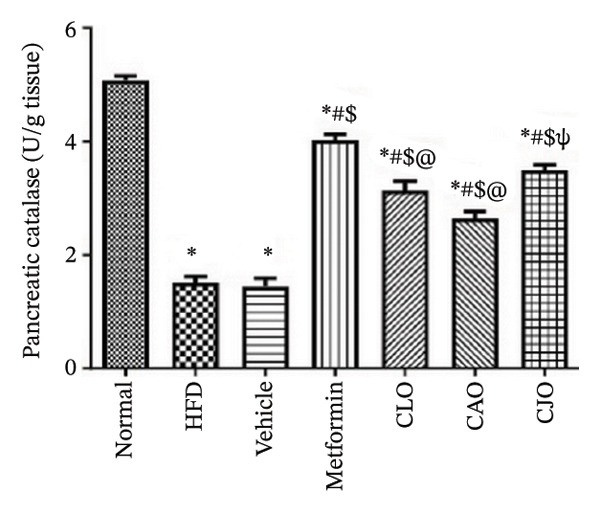
(c)

#### 3.3.4. Changes in Nrf2 and HO‐1 Expression in the Heart, Liver, and Pancreas in Obese Rats

The antioxidant Nrf2/HO‐1 system plays a critical role in maintaining the redox balance [37]. Herein, the gene expression of both Nrf2 (Figure 8) and HO‐1 (Figure 9) in the four examined tissues was significantly (*p* < 0.001) reduced in the HFD and vehicle groups compared to the normal control group. Meanwhile, oil emulsion groups showed significant rises in their expression levels compared to the HFD and vehicle groups. Clearly, the NE of CJO showed a more significant rise in the expression level of Nrf2 and HO‐1 in all studied tissues than the other oil treatment groups and also the metformin group. Notably, the effect of NE of CLO on both Nrf2 and HO‐1 expression levels was significantly higher than that of the CAO one.

FIGURE 8Effect of CLO (*Citrus limon*), CAO (*Citrus aurantiifolia*), and CJO (*Citrus japonica*) essential oils on nuclear factor‐erythroid 2‐related factor 2 (Nrf2) gene expression in (a) heart, (b) liver, and (c) pancreas in high‐fat diet (HFD)‐induced obesity in rats. Data are expressed as mean ± SEM. Significances are assigned as follows: ^∗^, compared to the normal control group; #, compared to the HFD group; $, compared to the vehicle group; @, compared to the metformin group; €, compared to the CLO group; ψ, the CAO group compared to the CJO group, using one‐way ANOVA followed by the Tukey–Kramer multiple comparison post hoc test.

**Figure:**
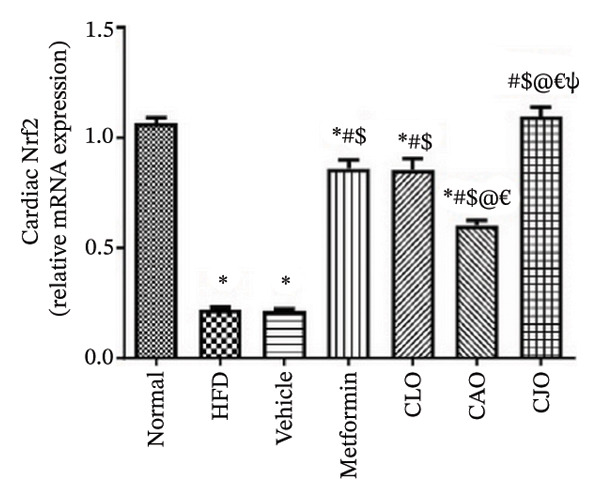
(a)

**Figure:**
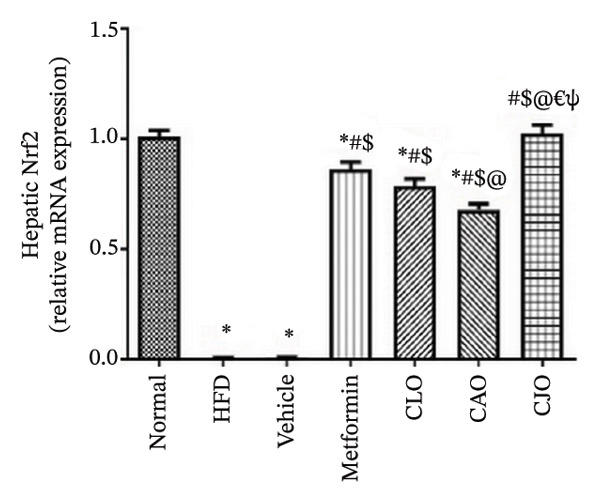
(b)

**Figure:**
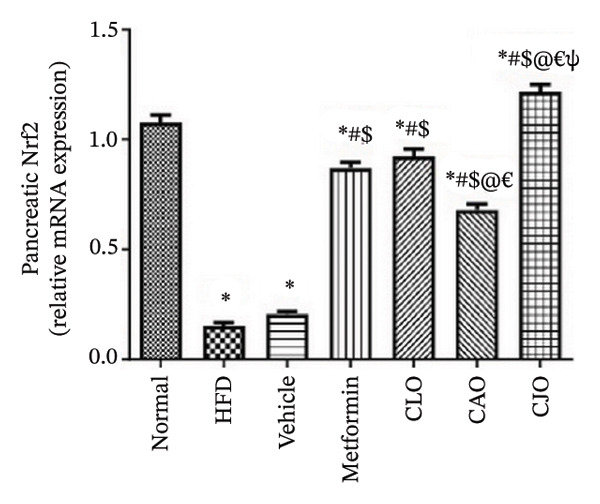
(c)

FIGURE 9Effect of CLO (*Citrus limon*), CAO (*Citrus aurantiifolia*), and CJO (*Citrus japonica*) on heme oxygenase‐1 (HO‐1) gene expression in (a) heart, (b) liver, and (c) pancreas in high‐fat diet (HFD)‐induced obesity in rats. Data are expressed as mean ± SEM. Significances are assigned as follows: ^∗^, compared to the normal control group; #, compared to the HFD group; $, compared to the vehicle group; @, compared to the metformin group; €, compared to the CLO group; ψ, the CAO group compared to the CJO group, using one‐way ANOVA followed by the Tukey–Kramer multiple comparison post hoc test.

**Figure:**
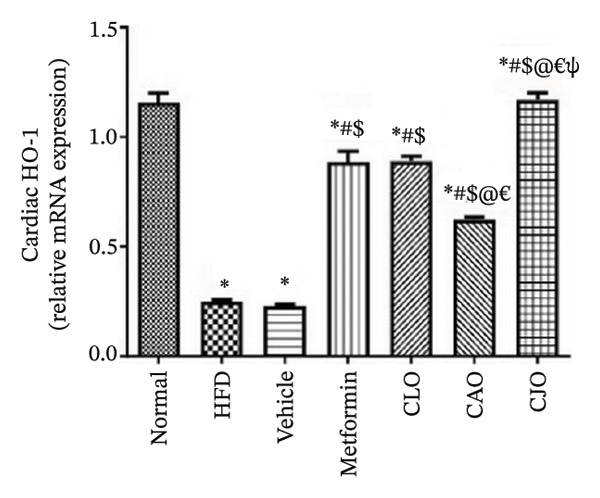
(a)

**Figure:**
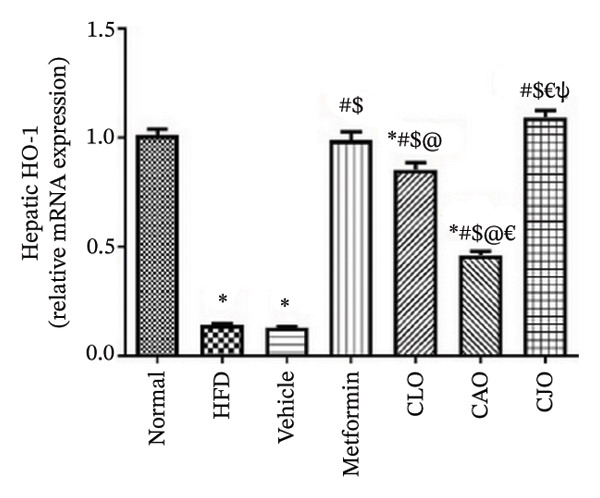
(b)

**Figure:**
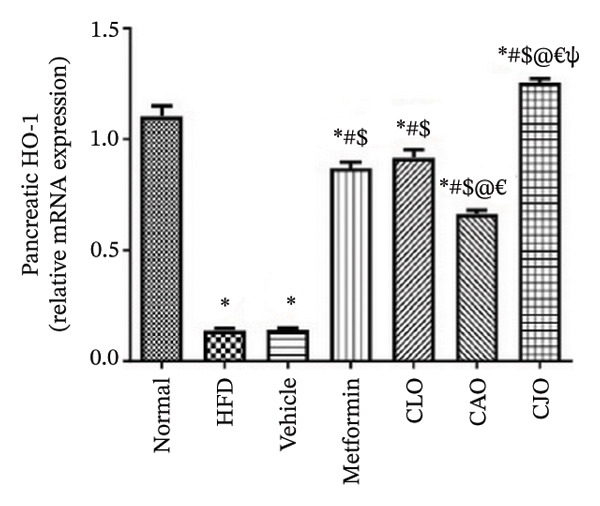
(c)

#### 3.3.5. Changes in Serum Adiponectin and Visfatin Levels in Obese Rats

Obesity as a predisposing factor of atherosclerosis is associated with reduced adiponectin levels [38]. Therefore, the HFD group showed a significant (*p* < 0.001) reduction in adiponectin levels (Figure 10(a)) when compared to the normal group. These levels were inversely and significantly (*p* < 0.001) elevated in all the treated groups with better effects of the CJO group than the other two NEs groups. On the other hand, the serum levels of visfatin, as a regulatory response to the HFD‐induced hyperglycemia [39], were significantly (*p* < 0.001) elevated in the HFD group when compared to the normal group (Figure 10(b)). Similarly, the CJO treatment showed a significantly superior reduction in visfatin levels than the other two NE treatments, with no significant effect compared to the metformin treatment.

FIGURE 10Effect of CLO (*Citrus limon*), CAO (*Citrus aurantiifolia*), and CJO (*Citrus japonica*) on serum (a) adiponectin and (b) visfatin in high‐fat diet (HFD)‐induced obesity in rats. Data are expressed as mean ± SEM. Significances are assigned as follows: ^∗^, compared to the normal control group; #, compared to the HFD group; $, compared to the vehicle group; @, compared to the metformin group; €, compared to the CLO group; ψ, the CAO group compared to the CJO group, using one‐way ANOVA followed by the Tukey–Kramer multiple comparison post hoc test.

**Figure:**
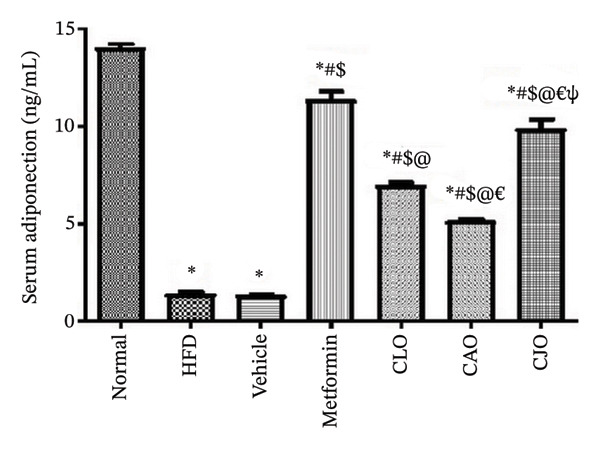
(a)

**Figure:**
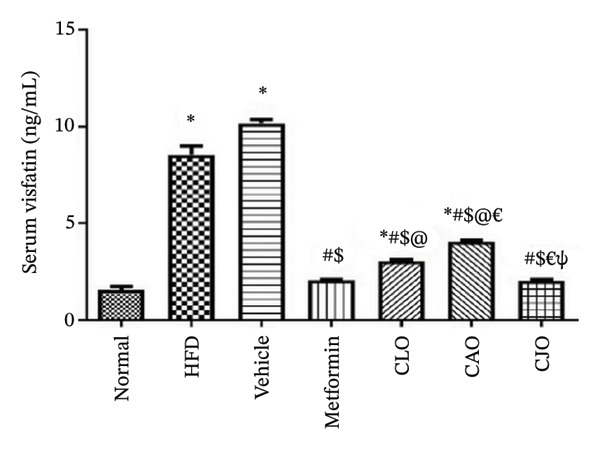
(b)

#### 3.3.6. Changes in SIRT1 Expression in the Heart, Liver, and Pancreas in Obese Rats

As shown in Figure 11(a), heart specimens of the normal control group showed moderate SIRT1 immunoexpression. In contrast, heart tissues of the HFD and vehicle groups were observed with minimal SIRT1 expression. Similar to the normal group, the metformin and the three oil‐treated groups showed moderate expression. Liver (Figure 11(c)) and pancreatic (Figure 11(e)) tissues were noticed with the same SIRT1 trend as the heart ones. For more illustration, the percentage of immunoexpression of SIRT1 in the heart, liver, and pancreas was represented in Figures 11(b), 11(d), and 11(f), respectively.

FIGURE 11Photomicrographs showing the effect of CLO (*Citrus limon*), CAO (*Citrus aurantiifolia*), and CJO (*Citrus japonica*) on immunoexpression of sirtuin 1 (SIRT1) in (a) heart, (c) liver, and (e) pancreas in high‐fat diet (HFD)‐induced obesity in rats. The normal control group has moderate SIRT1 expression. Meanwhile, the HFD and vehicle groups have very minimal expression of SIRT1. The metformin, CLO, CAO, and CJO groups show moderate SIRT1 expression (magnification 400x). (b), (d), and (f) corresponding percentages of SIRT1 immunoexpression in the heart, liver, and pancreas, respectively. Data are expressed as mean ± SEM. Significances are assigned as follows: ^∗^, compared to the normal control group; #, compared to the HFD group; $, compared to the vehicle group; @, compared to the metformin group; €, compared to the CLO group, using one‐way ANOVA followed by the Tukey–Kramer multiple comparison post hoc test.

**Figure:**
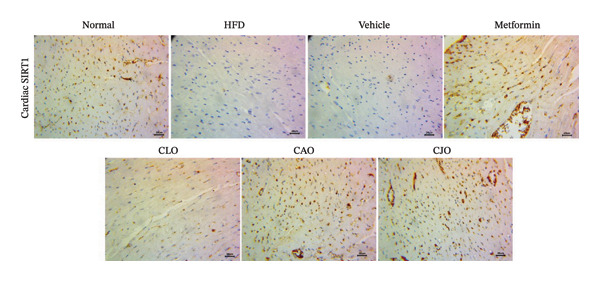
(a)

**Figure:**
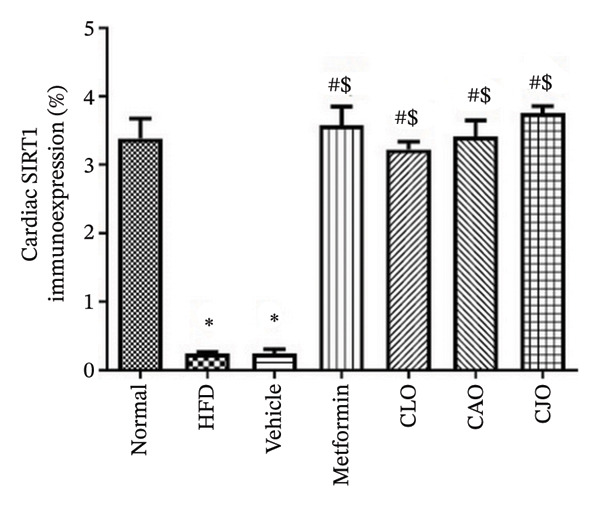
(b)

**Figure:**
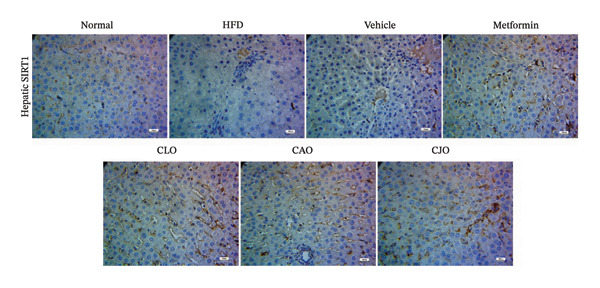
(c)

**Figure:**
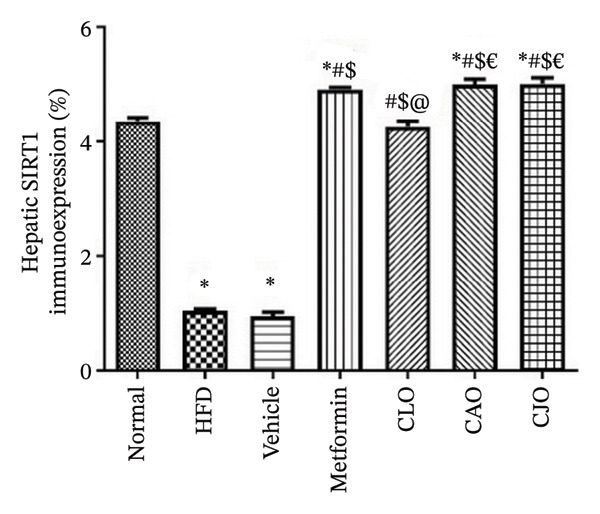
(d)

**Figure:**
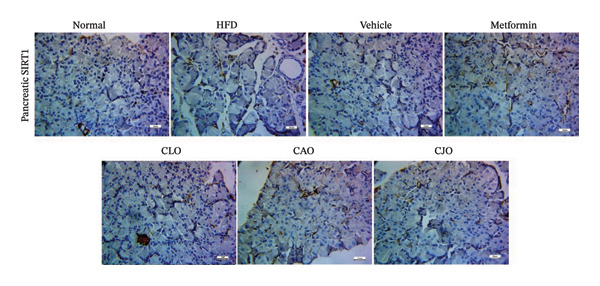
(e)

**Figure:**
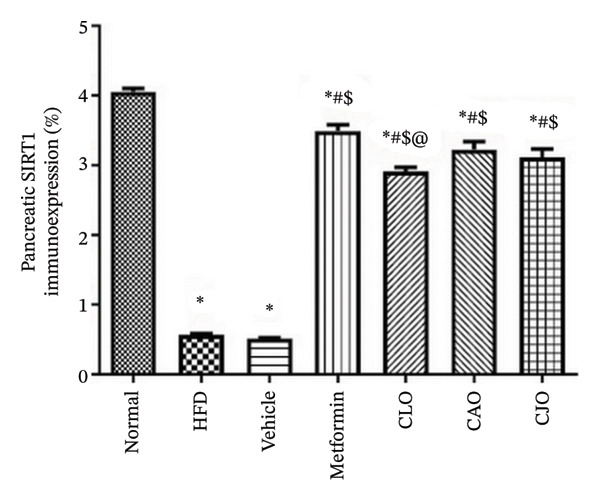
(f)

#### 3.3.7. Changes in Caspase‐3 Expression in the Heart, Liver, and Pancreas in Obese Rats

Heart, liver, and pancreatic tissue specimens of the normal group showed negative immunoexpression of active caspase‐3 (Figures 12(a), 12(c), and 12(e), respectively), while that of the HFD and vehicle groups had marked expressions. Clearly, the heart and pancreatic tissue specimens of the CJO group manifested minimal active caspase‐3 expression, while that of the liver of the same group was with negative expression as in the normal group. Moderate expressions were noticed in the three tissues of the CAO and CLO groups. For more illustration, the percentage immunoexpression of active caspase‐3 in the heart, liver, and pancreas was represented in Figures 12(b), 12(d), and 12(f), respectively.

FIGURE 12Photomicrographs showing the effect of CLO (*Citrus limon*), CAO (*Citrus aurantiifolia*), and CJO (*Citrus japonica*) essential oils on immunoexpression of active caspase‐3 in (a) heart, (c) liver, and (e) pancreas in high‐fat diet (HFD)‐induced obesity in rats. The normal control group has a negative expression of active caspase‐3 in the heart, liver, and pancreas. Meanwhile, the HFD and vehicle groups show marked expression of active caspase‐3 in the heart and liver and moderate expression in the pancreas. The metformin group has moderate expression of active caspase‐3 in the heart and minimal expression in the liver and pancreas. Otherwise, the CLO and CAO groups show minimal expression of active caspase‐3 that was very minimal in the heart, liver, and pancreas (magnification 400x). (b), (d), and (f) corresponding percentages of active caspase‐3 immunoexpression in the heart, liver, and pancreas, respectively. Data are expressed as mean ± SEM. Significances are assigned as follows: ^∗^, compared to the normal control group; #, compared to the HFD group; $, compared to the vehicle group; @, compared to the metformin group; €, compared to the CLO group; ψ, the CAO group compared to the CJO group, using one‐way ANOVA followed by the Tukey–Kramer multiple comparison post hoc test.

**Figure:**
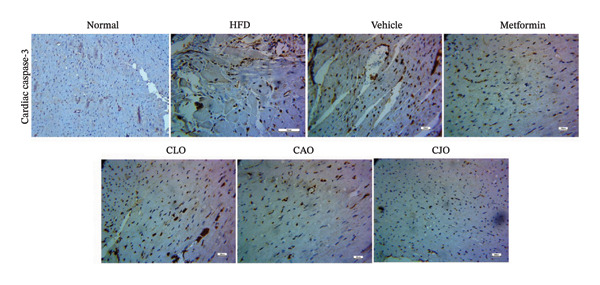
(a)

**Figure:**
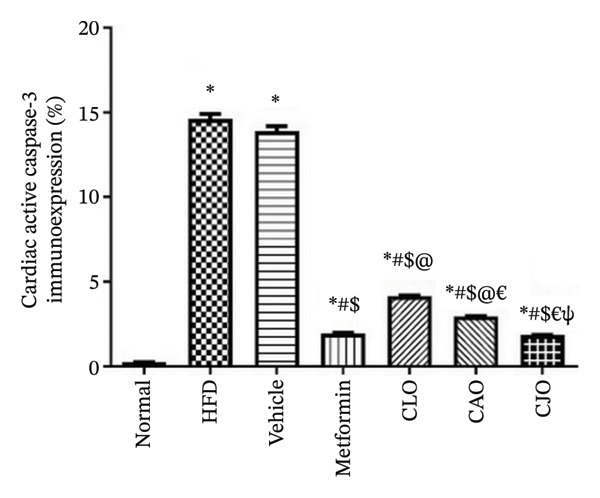
(b)

**Figure:**
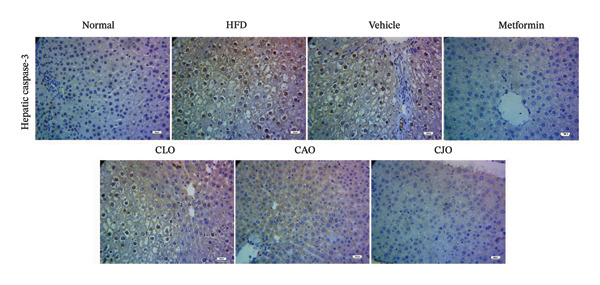
(c)

**Figure:**
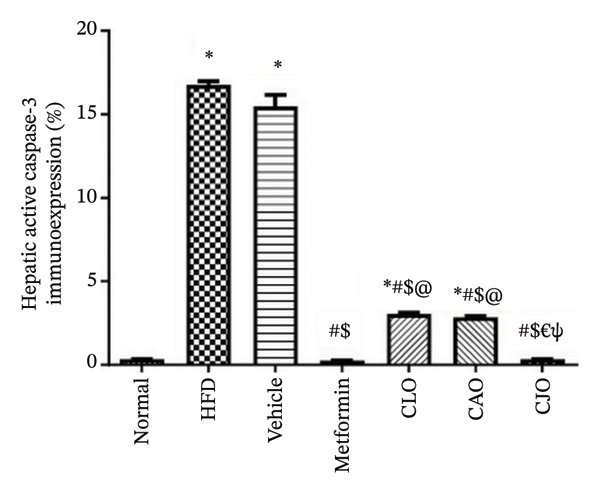
(d)

**Figure:**
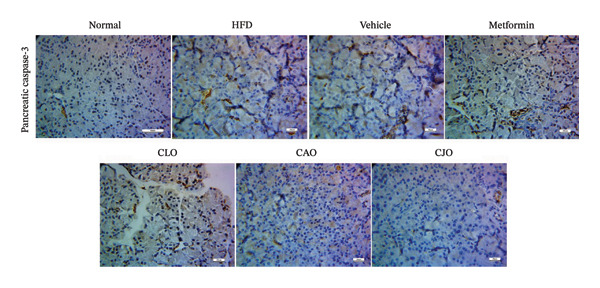
(e)

**Figure:**
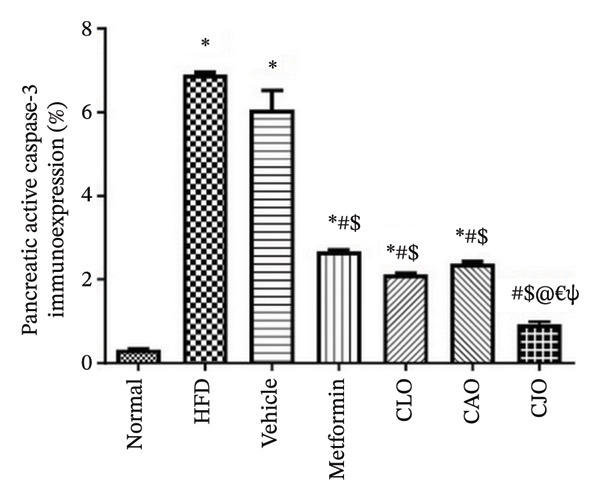
(f)

#### 3.3.8. Histopathological Changes of the Heart, Liver, and Pancreas in Obese Rats

As shown in Figure 13(a), H&E‐stained heart tissues of the normal control group revealed a normal structure of myocardium with regular arrangement of myocardial fibers and nuclei. Inversely, heart tissues of the HFD and vehicle groups showed irregularly arranged cardiomyocytes with wide spacing of cardiomyocytes, interstitial hemorrhage, and myocardial necrosis. Similar to the normal control group, heart specimens from other treated groups showed regular cardiomyocytes with minimal hemorrhage and necrosis. A semiquantitative scoring of cardiac histopathological lesions is demonstrated in Figure 13(b). Regarding liver tissues (Figure 13(c)), the normal group showed normal liver architecture and normal hepatocytes with normal radial arrangement around the central vein and portal triad without fat infiltration. In contrast, the liver specimen from the HFD and vehicle groups showed fatty vacuolization (coalesced fat droplets) with eccentric nuclei, area of focal necrosis, inflammatory infiltrate, and sinusoidal hemorrhages. The degree of fatty infiltrations, inflammatory infiltrates, and hemorrhage was minimal in liver specimens of the CJO group, while the other two oil groups (CLO and CAO) showed moderate fatty infiltration, inflammatory cell infiltrates, hemorrhage, and necrosis of hepatocytes. A semiquantitative scoring of hepatic histopathological lesions is demonstrated in Figure 13(d).

FIGURE 13Photomicrographs of H&E (hematoxylin and eosin)‐stained tissue specimens showing the effect of CLO (*Citrus limon*), CAO (*Citrus aurantiifolia*), and CJO (*Citrus japonica*) on histopathological changes in high‐fat diet (HFD)‐induced obesity in rats (magnification 400x). (a) The heart tissue: the normal control group has a regular arrangement of cardiomyocytes and nuclei. Meanwhile, the HFD and vehicle groups demonstrate disarranged swollen cardiomyocytes with deformed nuclei of cardiomyocytes and wide myocardial gap, as well as interstitial hemorrhage and necrosis. The metformin group presents regular cardiomyocytes with minimal deformed nuclei of cardiomyocytes. Otherwise, the CLO, CAO, and CJO groups show regular arrangement of cardiomyocytes with minor interstitial hemorrhage. (c) The liver tissue: the normal control group shows normal hepatocytes with no fat infiltration. Meanwhile, the HFD and vehicle groups have severe fatty infiltration with eccentric nuclei and areas of focal necrosis, inflammatory cell infiltrates, and sinusoid hemorrhage. The metformin group has minimal hemorrhage and fatty infiltrations. The CLO and CAO show moderate fatty infiltration, inflammatory cell infiltrates, and hemorrhage, and the CJO group shows mild fatty infiltration and nearly normal hepatocytes. (e) The pancreas tissue: the normal control group with typical pancreatic lobules are formed of crowded acini lined by pyramidal cells with basal vesicular nuclei, apical acidophilic zymogen granules, and basal basophilic cytoplasm. Meanwhile, the HFD and vehicle groups have disorganized pancreatic acini containing large deposits of intracellular hyaline material, many pyknotic nuclei in pyramidal acinar cells, and widened interlobular septa. The metformin group has a typical acinar structure with few pyknotic nuclei. Otherwise, the CLO and CAO groups show minimally degenerated pancreatic acini, and the CJO group demonstrates typical pancreatic acini. (b), (d), and (f) Corresponding histopathological scores in the heart, liver, and pancreas, respectively. Data are expressed as mean ± SEM. Significances are assigned as follows: ^∗^, compared to the normal control group; #, compared to the HFD group; $, compared to the vehicle group, using the nonparametric Kruskal–Wallis test.

**Figure:**
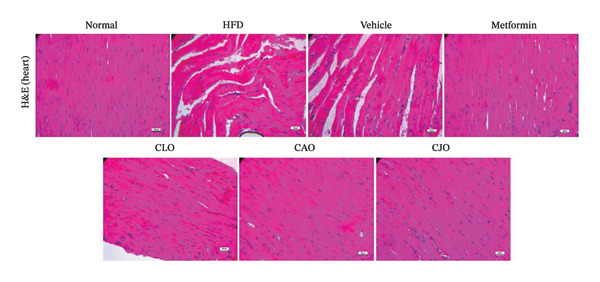
(a)

**Figure:**
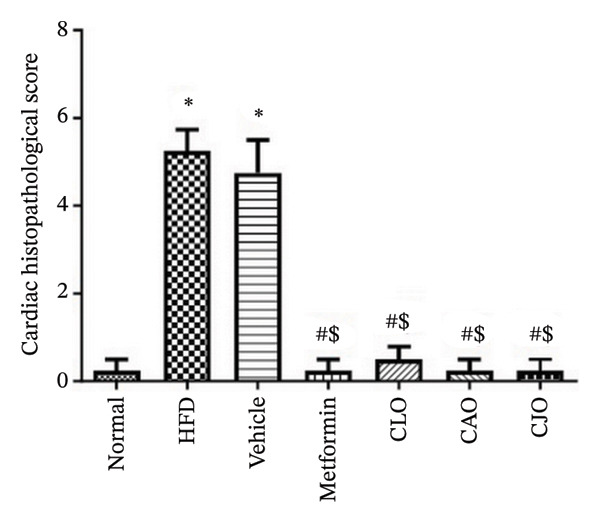
(b)

**Figure:**
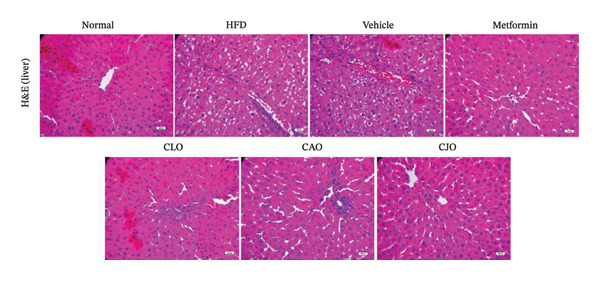
(c)

**Figure:**
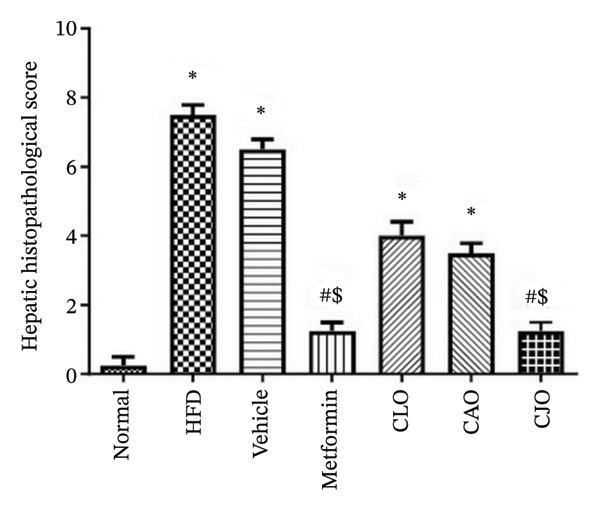
(d)

**Figure:**
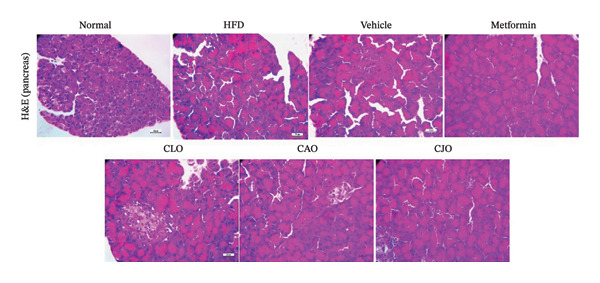
(e)

**Figure:**
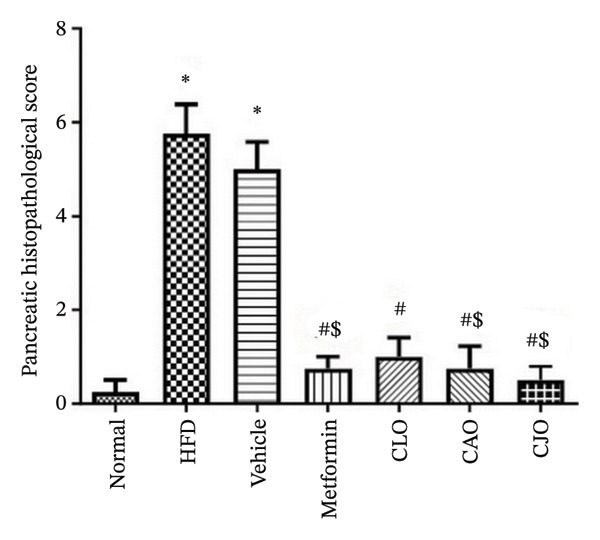
(f)

Figure 13(e) shows the H&E‐stained pancreatic specimens where the normal control group showed typical pancreatic lobules formed of crowded acini lined by pyramidal cells with basal vesicular nuclei, apical acidophilic zymogen granules, and basal basophilic cytoplasm. Meanwhile, the HFD and vehicle groups showed islet lesions in the form of shredded islets with irregular contour, reduced islet cells’ mass with vacuolated cytoplasm, pyknotic and karyolitic nuclei, and acinar lesions in the form of inflammatory cellular infiltrates and distorted acini with several pyknotic nuclei and wide interlobular septa were widened. Otherwise, pancreatic tissues of the three oil‐treated groups, specifically of the CJO group, showed minimal islet and acinar lesions. A semiquantitative scoring of pancreatic histopathological lesions is demonstrated in Figure 13(f).

## 4. Discussion

Obesity is linked to steatosis in various tissues, including the liver, heart, and pancreas, which can lead to different health issues such as cardiovascular morbidities, liver dysfunction, and pancreatic dysfunction. Currently, there is no pharmacological treatment approved for the management of obesity‐associated steatosis [40]. Accordingly, there is an urgent need to develop new therapeutic options to decrease the associated health consequences of obesity related to steatosis and to explore their underlying mechanisms. The current study was conducted to explore and compare the antisteatotic potential of the EOs of three *Citrus* species (*C. limon, C. aurantiifolia, and C. japonica*) in a rat model of steatosis associated with HFD‐induced obesity.

GC/MS analysis of the three EOs: CLO, CAO, and CJO, revealed 20, 28, and 35 compounds were identified, respectively. The nonoxygenated monoterpene, D‐limonene, is the major identified compound for both CLO (25.48%) and CAO (32.38%) but is present in trace amounts in CJO, while the oxygenated sesquiterpene, β‐eudesmol, is the predominant compound in CJO and absent in the other two oils. In CLO and CAO, monoterpenes represented 91.1% (48.22% of them were oxygenated) and 89.5% (52.16% of them were oxygenated), respectively, while, in CJO, it represented only 4.4% with the complete absence of oxygenated monoterpenes. In contrast, sesquiterpenes represented 3.32% and 7.56% in CLO and CAO, respectively, while in CJO, it represented a higher percentage of 89.78% (67.6% of them were oxygenated). Diterpenes were found only in CJO in a percentage of 3.18%. Although in this study the three oils were prepared from the species of the same genus, the chemical composition of CJO was completely different from the other two oils. Consistent with our results, previous studies showed that D‐limonene was the principal component in CLO and CAO [12, 41, 42].

In this study, HFD caused a significant rise in the body weight of rats, indicating the development of obesity. In addition, HFD caused a significant elevation in serum blood glucose, TGs, and total blood cholesterol, suggesting the impairment of glucose homeostasis and dyslipidemia. Therefore, HFD was considered a reliable experimental model of obesity [43]. Alternatively, the treatments with NEs of the three EOs significantly reduced the body weight, with a superior effect for the NE of CJO, which also showed a higher hypoglycemic effect comparable with metformin. Otherwise, NEs of the three EOs significantly reduced TGs; however, only the NE of CLO showed a significant lowering effect on total cholesterol, which was comparable with metformin. This suggests antiobesity, hypoglycemic, and hypolipidemic actions for these *Citrus* oil NEs with different potentialities.

The activity of CJO might be related to its sesquiterpene content, which represented a high percentage (89.78%) of its composition and was reported to exert an inhibitory activity against adipogenesis and obesity [44, 45]. On the other hand, D‐limonene, the principal component in CLO and CAO, was reported to be of special benefit in dyslipidemia and could help in the prevention of obesity [46, 47]. Compatible with these previous reports, this study showed that CLO and CAO, with their higher D‐limonene content, were more efficient in lowering total cholesterol level than CJO.

HFD is associated with dysfunction of different organs, including the heart, liver, and pancreas [48–50]. In the same context, HFD resulted in marked deteriorations in the functions of the investigated organs, including the heart, liver, and pancreas, as indicated by significant elevations of the serum function biomarkers CK‐MB, ALT, and CK, respectively, and further confirmed by associated histopathological changes. Interestingly, the treatment with the NEs of the three *Citrus* oils led to improvement in the functions and morphology of the heart, liver, and pancreas with different potentialities. In detail, cardiac functions were best improved by the NE of CAO, which presented better activity than metformin, whereas liver functions were remarkably ameliorated by the NE of CJO with a more pronounced effect than metformin. In the same way, the pancreatic function showed significant improvement only with the treatment with the NE of CJO, which was comparable with metformin.

Oxidative stress plays a crucial role in different organ damage in obesity [51, 52]. Cellular antioxidant responses are principally regulated by the transcription factor Nrf2, which controls the expression of different antioxidant genes, including mainly HO‐1[53]. The downregulation of the Nrf2/HO‐1 signaling is a coincidental feature of increased BMI [54]. In the current study, our findings revealed enhanced oxidative stress, evidenced by a significant rise in the levels of the lipid peroxidation biomarker, MDA, along with a significant reduction in the activities of the antioxidant enzymes, GSH and CAT, that were correlated with the significantly lower genetic expression of Nrf2 and HO‐1 in the heart, liver, and pancreas of the HFD group. Successfully, treatments with CJO, CLO, and CAO, in decreasing order, attenuated the oxidative stress with the upregulation of the cellular antioxidant enzymes GSH and CAT and the expression of Nrf2 and HO‐1 genes, highlighting that antioxidant potentials could be a possible mechanism of the cytoprotective effects of these oils against the hazardous consequences of obesity. Consistent with these findings, the EOs of *C. limon*, *C. aurantiifolia,* and *C. japonica* exhibited free radical scavenging activities in different experimental studies evaluating the antioxidant activities [55–57].

Oxidative stress in accumulated fat impairs the regulation of adipocytokine production, which has been associated with obesity/overweight and obesity‐associated metabolic syndrome. Dysregulated adipocytokines are mainly represented by decreased adiponectin and increased visfatin plasma levels [4, 58]. Adiponectin is an anti‐inflammatory adipocytokine that regulates a number of metabolic pathways involved in glucose and lipid homeostasis [59]. On the other hand, visfatin has been associated with oxidative stress and inflammation as well as the modulation of several genes involved in lipid homeostasis [39, 60, 61]. Herein, our data outlined that HFD resulted in a significant reduction in adiponectin levels and a rise in visfatin levels. On the other side, the treatments with the three NEs resulted in a significant elevation of serum levels of adiponectin with a significant reduction in serum visfatin, with the NE of CJO being the most effective one, showing comparable effects with metformin. These findings suggest that the modulatory effects of the three *Citrus* oils on adipocytokine levels underlie their beneficial effects on HFD‐induced obesity‐associated steatosis. According to a different study by Ref. [62], EO of *C. limon* enhanced lipolysis and suppressed body weight growth by activating the sympathetic nerve activity that innervates white adipose tissue, where adiponectin is released.

Numerous connections between the energy metabolism and inflammation and sirtuins (SIRT1‐SIRT7) protein family have been established in several studies [63–65]. Meaningly, it has been postulated that pharmacological activators of SIRT1 can increase mitochondrial capacity, enhance whole‐body glucose homeostasis after a high‐fat meal [66], and elevate the level of different antioxidant enzymes [67]. Moreover, SIRTs have been addressed as among the cellular modulators of adipocytokine‐mediated metabolic modulation [68]. Recently, a link between obesity and downregulation of SIRT1 expression has further been reported [69]. In addition, SIRT1 has other cellular protective roles, including suppression of oxidative stress via mediating Nrf2 activation and potentiation of its downstream antioxidant genes as well as inhibiting apoptotic response associated with oxidative stress and inflammation [70–72]. In this study, a significant reduction in the expression of SIRT1 in liver, pancreas, and heart of the HFD group was noticed while treatments with the NEs stimulated a significant rise in SIRT1 expression in different examined tissues with the NE of CJO being the most prominent one. These results were consistent with the observed upregulation of the antioxidant genes, Nrf2 and HO‐1, by the three NEs and collectively could explain their protective effect against organ steatosis.

Previous studies confirmed that obesity‐induced tissue damage in rats and mice manifested apoptosis and upregulation of the apoptotic executor, caspase‐3 [73–78]. According to these previous reports, we found a significant rise in the expression of caspase‐3 in the heart, liver, and pancreas in HFD‐fed rats. Inversely, treatments with the three NEs in this experiment promoted a significant reduction of caspase‐3 expression, especially with the NE of CJO, suggesting that the inhibition of apoptosis might be an additional mechanism for their cytoprotective potential. In conjunction, pure EOs inhibited heat shock‐induced apoptosis and blocked the activation of caspase‐3 in the human astrocyte cell line CCF‐STTG1 [79].

The present study has some limitations, which include the absence of direct myocardial and pancreatic lipid quantification (e.g., Oil Red O staining). Future studies incorporating direct lipid assessment are warranted to provide definitive evidence of tissue‐specific steatosis. Moreover, mechanistic studies, through pharmacological inhibition or genetic manipulation, are warranted to assert the direct role of adiponectin/SIRT1/Nrf2 signaling pathway in the protective effects of the three *Citrus* EOs. A non‐nano‐EO comparator is also needed for definitive conclusions regarding NE‐specific advantages.

## 5. Conclusion

**FIGURE 14:**
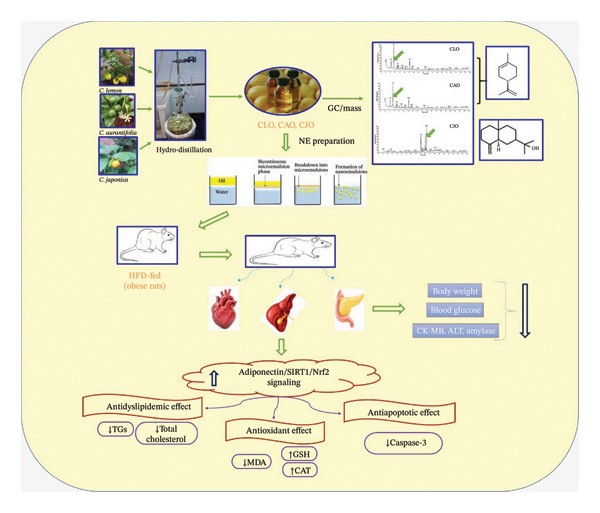
Schematic representation of the current study showing the beneficial effects of the nanoemulsions of the citrus essential oils of *Citrus limon* (CLO), *Citrus aurantiifolia* (CAO), and *Citrus japonica* (CJO) on cardiac, hepatic, and pancreatic steatosis in obese rats (↑: increase; ↓: decrease, CK‐MB: creatine kinase‐myocardial band; ALT: alanine aminotransferase; SIRT1: sirtuin 1; Nrf2: nuclear factor‐erythroid 2‐related factor 2; TGs: triglycerides; MDA: malondialdehyde; GSH: reduced glutathione; CAT: catalase).

The current study indicates that treatments with the three *Citrus* EOs (CLO, CAO, and CJO) in the form of NEs have significant ameliorative effects on obesity‐induced hepatic steatosis and cardiac and pancreatic injury in rats through hypoglycemic, antidyslipidemic, cytoprotective, antioxidant, and antiapoptotic activities. These beneficial effects are collectively associated with modulating adiponectin/SIRT1/Nrf2 signaling pathway (Figure 14). Among the three NEs, the CJO (150 mg/kg) one had the strongest antisteatosis effect in the liver, heart, and pancreas of obese rats, which was more potent than standard metformin (500 mg/kg), especially regarding the antioxidant activity.

## Author Contributions

Conception or design: Amira Mira, Yhiya Amen, Abdelaziz M. Hussein, Dalia Shaheen, Elsayed A. Eid, and Amal F. Soliman.

Acquisition, analysis, or interpretation of data: Amira Mira, Yhiya Amen, Abdelaziz M. Hussein, Abdullah Haikal, Mahmoud Elshal, Dalia Shaheen, Elsayed A. Eid, Ahmed Magdy Aly, Mennatullah A. M. Hussein, Abdelrahman H. K. Moustafa, Amgad E. Salem, and Amal F. Soliman.

Drafting the work or revising: Amira Mira, Yhiya Amen, Abdelaziz M. Hussein, Abdullah Haikal, Mahmoud Elshal, Dalia Shaheen, Elsayed A. Eid.

Final approval of the manuscript: Amira Mira, Yhiya Amen, Abdelaziz M. Hussein, Abdullah Haikal, Mahmoud Elshal, Dalia Shaheen, Elsayed A. Eid, and Amal F. Soliman.

## Funding

No funding was received for this manuscript.

## Conflicts of Interest

The authors declare no conflicts of interest.

## Supporting Information

Additional supporting information can be found online in the Supporting Information section.

## Supporting information


**Supporting Information** Supporting tables show the compositions and active ingredients in the three essential oils studied. Bold values in tables point out the major components. Table S1 shows the composition of C. *limon* aerial parts. Table S2 shows the composition of C. *aurantiifolia* aerial parts. Table S3 shows the composition of *C. japonica* aerial parts.

## Data Availability

The data that support the findings of this study are available from the corresponding author upon reasonable request.
