# Supplementary material for: Citrus Essential Oil Nanoemulsions Mitigate Cardiac, Hepatic, and Pancreatic Injury in High‐Fat Obese Rats via Adiponectin/SIRT1/Nrf2 Signaling Pathways
Source: J Nutr Metab. 2026 May 30;2026:3343221. doi: 10.1155/jnme/3343221 (PMC13240386; doi:10.1155/jnme/3343221)
Supplement: Supplementary file 1 — Supporting Information Supporting tables show the compositions and active ingredients in the three essential oils studied. Bold values in tables point out the major components. Table S1 shows the composition of C. limon aerial parts. Table S2 shows the composition of C. aurantiifolia aerial parts. Table S3 shows the composition of C. japonica aerial parts. [file JNME-2026-3343221-s001.doc]

**Table S1: Essential oils’ composition of *C. limon* aerial parts.**

| **Peak No.** | **Retention time** | **Literature R*_I_*** | **Calculated R*_I_*** | **M^+^ peak** | **Base peak** | **Peak area %** | **Identified compounds** |
| --- | --- | --- | --- | --- | --- | --- | --- |
| 1 | 2.03 | 841 | 839 | 84 | 56 | 4.57 | 3-Methylpentanol |
| 2 | 3.67 | 939 | 938 | 136 | 93 | 1.25 | *α*- Pinene |
| 3 | 4.31 | 975 | 977 | 136 | 93 | **8.43** | **Sabinene** |
| 4 | 4.41 | 979 | 980 | 136 | 93 | 1.14 | *β*-Pinene |
| 5 | 5.32 | 1026 | 1025 | 134 | 119 | 2.21 | *O*-Cymene |
| 6 | 5.41 | 1029 | 1031 | 136 | 68 | **25.48** | D-Limonene |
| 7 | 6.89 | 1050 | 1053 | 136 | 93 | 4.37 | *β*- Ocimine |
| 8 | 7.05 | 1096 | 1096 | 154 | 71 | **7.60** | Linalool |
| 9 | 7.44 | 1102 | 1099 | 152 | 81 | 5.49 | *Cis*- Thujone |
| 10 | 8.28 | 1153 | 1151 | 154 | 69 | 4.14 | Citronellal |
| 11 | 9.06 | 1164 | 1166 | 150 | 108 | 5.03 | Terpinen-4-ol |
| 12 | 9.40 | 1177 | 1179 | 154 | 71 | 3.04 | Pinocarvone |
| 13 | 10.10 | 1216 | 1214 | 152 | 109 | 1.93 | Carveol |
| 14 | 10.26 | 1225 | 1227 | 156 | 69 | 2.07 | Citronellol |
| 15 | 10.66 | 1243 | 1241 | 150 | 82 | 2.80 | Carvone |
| 16 | 11.45 | 1267 | 1266 | 152 | 69 | 1.83 | Geranial |
| 17 | 12.22 | 1286 | 1289 | 154 | 71 | **10.85** | **5 - Caranol** |
| 18 | 13.40 | 1321 | 1323 | 170 | 71 | 3.44 | Limonene-1,2-diol |
| 19 | 18.85 | 1578 | 1581 | 220 | 43 | 0.95 | Spathulenol |
| 20 | 18.92 | 1585 | 1586 | 220 | 43 | 2.37 | Caryophyllene oxide |
|  |  |  |  |  |  | 98.99 |  |

**Bold values**: point out the major components.

**Table S2: Essential oils’ composition of *C. aurantiifolia* aerial parts.**

| **Peak No.** | **Retention time** | **Literature R*_I_*** | **Calculated R*_I_*** | **M^+^ peak** | **Base peak** | **Peak area %** | **Identified compounds** |
| --- | --- | --- | --- | --- | --- | --- | --- |
| 1 | 2.03 | 841 | 839 | 84 | 56 | 1.72 | 3-Methylpentanol |
| 2 | 3.67 | 939 | 938 | 136 | 93 | 1.12 | *α*-Pinene |
| 3 | 4.31 | 975 | 977 | 136 | 93 | 2.17 | Sabinene |
| 4 | 4.41 | 979 | 980 | 136 | 93 | 0.79 | *β*-Pinene |
| 5 | 4.58 | 990 | 989 | 136 | 93 | 0.88 | *β*-Myrcene |
| 6 | 5.41 | 1029 | 1031 | 136 | 68 | **32.38** | D-Limonene |
| 7 | 7.05 | 1096 | 1096 | 154 | 71 | 2.01 | Linalool |
| 8 | 8.28 | 1153 | 1151 | 154 | 69 | 1.47 | Citronellal |
| 9 | 9.06 | 1168 | 1171 | 136 | 43 | 1.19 | Trans-4-Thujanol |
| 10 | 10.10 | 1216 | 1214 | 152 | 109 | 1.19 | Carveol |
| 11 | 10.20 | 1229 | 1231 | 154 | 41 | 2.09 | Nerol |
| 12 | 10.50 | 1238 | 1237 | 152 | 41 | **9.52** | **Neral** |
| 13 | 10.66 | 1243 | 1241 | 150 | 82 | 1.41 | Carvone |
| 14 | 11.27 | 1252 | 1252 | 154 | 69 | **12.92** | **Geraniol** |
| 15 | 11.45 | 1267 | 1266 | 152 | 69 | 2.59 | Geranial |
| 16 | 12.22 | 1286 | 1289 | 154 | 71 | 1.57 | 5-Caranol |
| 17 | 12.92 | 1288 | 1291 | 197 | 43 | 4.64 | Trans- linalool oxide acetate |
| 18 | 13.40 | 1321 | 1323 | 170 | 71 | 4.62 | Limonene-1,2-diol |
| 19 | 13.53 | 1361 | 1359 | 154 | 69 | 2.17 | Neryl acetate |
| 20 | 14.06 | 1381 | 1383 | 154 | 69 | 2.42 | Geranyl acetate |
| 21 | 14.34 | 1390 | 1390 | 204 | 93 | 1.05 | *β*-Elemene |
| 22 | 15.53 | 1455 | 1456 | 194 | 43 | 1.06 | Geranyl acetone |
| 23 | 15.68 | 1566 | 1567 | 182 | 69 | 1.29 | Methyl geranate |
| 24 | 18.92 | 1585 | 1586 | 220 | 43 | 3.36 | Caryophyllene oxide |
| 25 | 19.56 | 1599 | 1603 | 222 | 43 | 0.92 | Widdrol |
| 26 | 20.04 | 1608 | 1610 | 220 | 43 | 1.14 | Humulene oxide II |
| 27 | 20.27 | 1623 | 1621 | 220 | 43 | 1.09 | Isospathulenol |
| 28 | 24.16 | 1681 | 1678 | 198 | 85 | 1.12 | *γ*-Dodecalactone |
|  |  |  |  |  |  | 99.9 |  |

**Bold values**: point out the major components.

**Table S3: Essential oils’ composition of *C. japonica* aerial parts.**

| **Peak No.** | **Retention time** | **Literature R*_I_*** | **Calculated R*_I_*** | **M^+^ peak** | **Base peak** | **Peak area %** | **Identified compounds** |
| --- | --- | --- | --- | --- | --- | --- | --- |
| 1 | 2.03 | 841 | 839 | 84 | 56 | 1.46 | 3-Methylpentanol |
| 2 | 5.32 | 1026 | 1025 | 134 | 119 | 1.99 | O-Cymene |
| 3 | 5.41 | 1029 | 1031 | 136 | 68 | 1.22 | D-Limonene |
| 4 | 11.51 | 1298 | 1301 | 128 | 128 | 1.19 | Azulene |
| 5 | 12.95 | 1338 | 1339 | 204 | 121 | 1.04 | *δ*-EIemene |
| 6 | 14.34 | 1390 | 1390 | 204 | 93 | 1.35 | *β*-Elemene |
| 7 | 15.06 | 1419 | 1416 | 204 | 91 | 1.03 | Caryophyllene |
| 8 | 15.66 | 1420 | 1419 | 204 | 161 | 1.33 | *β*-Cedrene |
| 9 | 16.52 | 1438 | 1435 | 204 | 81 | 0.93 | Humulene |
| 10 | 16.74 | 1441 | 1444 | 204 | 41 | 1.11 | Aromadendrene |
| 11 | 17.41 | 1460 | 1459 | 204 | 41 | 0.86 | Alloaromadendrene |
| 12 | 17.73 | 1488 | 1488 | 192 | 177 | 0.87 | *β*-Ionone |
| 13 | 17.95 | 1488 | 1489 | 204 | 105 | 0.86 | Aristolochene |
| 14 | 18.21 | 1490 | 1491 | 204 | 105 | **12.79** | *β*-Selinene |
| 15 | 18.37 | 1500 | 1501 | 204 | 105 | 0.88 | *α*-Muurolene |
| 16 | 18.50 | 1503 | 1505 | 222 | 207 | 3.14 | *β*-Dihydroagarofurane |
| 17 | 18.60 | 1549 | 1550 | 204 | 59 | 0.83 | Elemol |
| 18 | 18.74 | 1563 | 1561 | 204 | 41 | 1.25 | E-Nerolidol |
| 19 | 18.85 | 1578 | 1581 | 220 | 43 | 2.98 | Spathulenol |
| 20 | 18.92 | 1585 | 1586 | 220 | 43 | 2.18 | Caryophyllene oxide |
| 21 | 19.10 | 1591 | 1595 | 222 | 109 | 1.54 | Globulol |
| 22 | 19.28 | 1607 | 1609 | 220 | 43 | 3.82 | *β*-Oplopanone |
| 23 | 19.56 | 1632 | 1633 | 222 | 189 | 1.25 | *γ*-Eudesmol |
| 24 | 20.06 | 1641 | 1637 | 220 | 41 | 5.13 | Alloaromadendrene epoxide |
| 25 | 20.15 | 1641 | 1639 | 222 | 161 | **12.62** | **Hinesol** |
| 26 | 20.62 | 1650 | 1649 | 222 | 59 | **20.25** | ***β*-Eudesmol** |
| 27 | 22.33 | 1662 | 1663 | 206 | 59 | 1.60 | Dihydro-Eudesmol |
| 28 | 23.13 | 1685 | 1686 | 218 | 43 | 1.21 | Bisabolene oxide A |
| 29 | 23.33 | 1700 | 1702 | 222 | 43 | 1.14 | Eudesm-7(11)-en-4-ol; Juniper camphor |
| 30 | 23.76 | 1763 | 1766 | 218 | 41 | 1.79 | Aristolone |
| 31 | 24.14 | 1843 | 1845 | 218 | 185 | 1.66 | *α*-Vetivone |
| 32 | 24.35 | 1897 | 1901 | 220 | 149 | 2.75 | Cedran-diol, 8S,13- |
| 33 | 25.07 | 1927 | 1925 | 218 | 59 | 2.46 | Carissone |
| 34 | 25.27 | 1943 | 1944 | 278 | 71 | 1.72 | Phytol |
| 35 | 25.87 | 2198 | 2202 | 284 | 91 | 1.46 | Retinal |
|  |  |  |  |  |  | 99.69 |  |

**Bold values**: point out the major components.
